# 

**Published:** 1884-12-20

**Authors:** 


### TABLE OF CONTENTS.

ORIGINAL AND SELECTED ARTICLES:
Epithelioma, by H. M. Lawson, M. D., Cuthbert, Ga., 441. Red Clay as a Remedy for Sprains, by G. G. Roy, M. D., Professor of Materia Medica in the Southern Medical College, Atlanta, Ga., 444. Malarial Hæmorrhagic Fever, by J. K. Hodge, M. D., of Princeton, Ark., 445. Obstruction of Ureter—Its Consequences and Remedy, by J. McF. Gaston, M. D., Professor of Principles and Practice of Surgery in the Southern Medical College, Atlanta, Ga., 447. A Case of Imperforate Rectum in which Lumbar Colotomy was performed, by John H. Packard, M. D., Surgeon to the Pennsylvania Hospital and to St. Joseph's Hospital, Philadelphia, 452 Cholera Researches of Dr. Koch, translated from the Courier des Etats Unis by S. Pollak, M. D., St. Louis, 455. Notes on a case of Poisoning from Mrs. Winslow's Soothing Syrup, read before the Philadelphia County Medical Society, by A. B. Hirsh, M. D., 456.

ABSTRACTS AND GLEANINGS:
Case of Dropsy cured, 458. Experiences with Hydrochlorate of Cocoaine, 458. The effects of Bromoform, and Bromethyline, 459. Some statistics of Abdominal Surgery, 460. Foreign bodies in the Air-passages, 561 Substitute for Veratrum, 462. Hemorrhoids, Dilatation of Anus for, 462. My Child's Orign, 463 The Dry Diet treatment of Ulcer of the Stomach, 463. Chronic dysentery, 464. The medicines physicians use, 464. Crede's method of Expression of the Placenta, 465. Numbness of Upper Extremities, 465. Salicylic Acid and Spirits Nitre as a Febrifuge, 466. Emenagogue, 466. A decoction of Lemon as a cure for Ague, 466. Pieces of glass pass through the digestive tract, 467. Diphtheria and Croup, 468. A new preparation of Mercury, 468. Iodide of Starch in treatment of Intermittent Fever, 468. Morphia hypodermic injections for Infantile Convulsions, 468. The International Medical Congress for 1887, 469. Nitrite of Amyl, 469. Electric treatment of Perimetritis, 469.

SCIENTIFIC ITEMS:

Tempest in the Polar Seas, 470; Effects of lightning stroke on human beings, 470; Tanning leather by electricity, 471.

PRACTICAL NOTES AND FORMULÆ.
Gonorrhœa easily cured; Boils; Golden Eye Water, 472. Picrate of Ammonia in Whooping Cough; Enlarged Tonsils; Acute Alcoholism; Nervous Headache; Ague Cure, 473.

EDITORIALS AND MISCELLANEOUS.
To Our Subscribers; Our Journal for 1885; Editor'al Notices, 475; Dr. J. Thad. Johnson; Liberality of a Macon Drug House; Goodwyn's Compound Syrup of the Hypophosphites; Salary of the Atlanta City Doctors; Cancer, 476; Chicago Medical Society, 477; Books and Pamphlets received, 478; Receipts; Special Notices, 480.

